# Assessment of the Foot’s Longitudinal Arch by Different Indicators and Their Correlation with the Foot Loading Paradigm in School-Aged Children: A Cross Sectional Study

**DOI:** 10.3390/ijerph18105196

**Published:** 2021-05-13

**Authors:** Beata Szczepanowska-Wołowiec, Paulina Sztandera, Ireneusz Kotela, Marek Zak

**Affiliations:** 1 Institute of Health Sciences, Collegium Medicum, Jan Kochanowski University, Zeromskiego 5, 25-369 Kielce, Poland; beatawolowiec@op.pl (B.S.-W.); sztandera.paulina@gmail.com (P.S.); 2 Rehabilitation Clinic, Provincial General Hospital, 25-310 Kielce, Poland; 3 Institute of Medical Sciences, Collegium Medicum, Jan Kochanowski University, Zeromskiego 5, 25-369 Kielce, Poland; ikotela@op.pl; 4 Central Clinical Hospital of the MSWiA, 02-507 Warsaw, Poland

**Keywords:** foot, longitudinal arches, feet deformities, podology, public health

## Abstract

Background: There are numerous studies assessing the morphological structure of the foot, but there is a notable scarcity of those focused on juxtaposing various longitudinal arch indices with foot loading paradigm. The present study aimed to determine the overall reliability, diagnostic accuracy of respective variables, and their correlation with the foot loading paradigm. Methods: The study group consisted of 336 children, aged 10–15 years (girls 49.1% and boys 50.9%). The morphological structure of the plantar part of the foot in static conditions was assessed with the aid of a 2D podoscan. Individual foot loading paradigm in static conditions was assessed making use of the FreeMed platform. Results: Staheli (SI), Chippaux–Smirak (CSI), and Sztriter–Godunow (KY) indices were strongly correlated with each other (ρ > 0.84, *p* < 0.001). Own research corroborated an increased pressure of hollow feet, as assessed by the SI, CSI, and KY indices, on the forefoot and the hindfoot, foot zones B, E, F; these correlations being statistically significant. The results yielded by the present study also indicate an increased pressure on the metatarsal, and foot zones C, D of the flat feet. Conclusions: Flatfootedness is not believed to be a common deformity among children and adolescents. The SI, CSI, and KY indices were found to be strongly correlated, as well as proved reliable in assessing the foot’s longitudinal arch.

## 1. Introduction

A human foot makes an essential, comprehensively structured component of support, locomotion, and shock absorption. The consequences of any abnormalities within the foot structure may exert long-term effects, as well as affect the overall functionality of the joints in the lower limbs, and consequently of the entire body frame [1,2].

Effective locomotion and load-bearing function serving the entire body frame owes its technical feasibility to overall mobility and the high extent of the foot’s flexibility. Overall mechanics of the foot is dependent upon the structure of its arches. The medial longitudinal arch (a dynamic one) runs from the calcaneus to the head of the first metatarsal bone. It is formed by the ankle bone, the scaphoid bone, the cuneiform bone, and the metatarsal bone. The lateral longitudinal arch is formed by the heel bone, the cuboid bone, and the fourth and fifth metatarsal bones. The foot also features a transverse arch at the level of the cuboid bone, the cuneiform bone, and the bases of all the metatarsal bones. The special shape of the bones and the complex musculoskeletal system of the foot allows these arches to be maintained [1].

**Figure 1:**
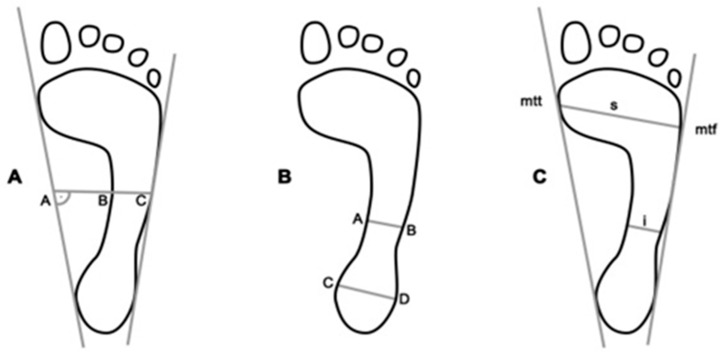
The manner of determining respective indicators. Source: own research material. (**A**) Sztriter-Godunow index (KY); (**B**) Staheli index (SI); (**C**) Chippaux-Smiraka index (CSI).

A change in the medial longitudinal arch (MLA) of the foot may bring about a cascade of implications for the function of the entire musculoskeletal system [3,4]. The height of the longitudinal arch impinges upon the alignment of the lower limb. An elevated longitudinal arch affects the foot’s supination alignment, whereas a lowered one is associated with its pronation [2,4]. Altering the height of the MLA has been identified as a significant cause of lower limb injury [3]. Many techniques can be used to assess the medial longitudinal arch of the MLA, i.e., indirect and direct ones. Indirect methods comprise the ink-marked or digital tracing (static, dynamic), and a variety of photographic techniques. The direct methods comprise somatometric measurements, clinical assessment, radiographic, and ultrasound assessment. Tracing is by far the most commonly applied method in assessing MLA. In line with this technique, MLA may be measured by making use of different types of indices, e.g., the Clarke’s angle (footprint angle), Chippaux–Simirak index (CSI), Sztriter–Godunow index (KY), and Staheli index (SI) (Figure 1) [4,5,6].

The diagnostics of any foot defects are usually supported by a fully-fledged clinical examination. The actual subjectivity of such an examination has promoted the development of a number of methods aimed at having the accuracy of such a diagnosis effectively confirmed. As there is no standardized protocol for examining the longitudinal arch, the application of different assessment methods for tracing the imprint of the plantar part of the foot accounts for contradictory conclusions with regard to the actual incidence of various foot deformities among children and adolescents. As far as routine clinical practice is concerned, there is a notable need for an easy, sensitive, and highly reliable method meant to have each diagnosis effectively verified and ultimately confirmed. Accurate classification and diagnosis of the foot is essential, as it may then be taken as the starting point for mapping out the target-oriented, treatment management plan. Whilst assessing the foot’s longitudinal arch by applying the SI, CSI, and KY indices, in conjunction with the percentage of foot loading on its respective zones, an attempt was made at establishing conclusively which of the above-referenced indices boasted by far the greatest diagnostic potential.

The study principally aimed at establishing overall reliability and diagnostic accuracy of respective, select morphological variables of the foot, and their correlation with an individual foot loading paradigm. The findings may well be of essential significance in foot diagnostics and overall prevention policy making, apart from aiding a pragmatic selection of the indicators best suited for a reliable assessment of the foot’s morphological structure.

## 2. Methods

### 2.1. The Study Subjects

The survey involved 336 children (165 girls—49.1%, and 171 boys—50.9%; aged 10–15 years), randomly selected from primary schools, whose characteristics are presented in Table 1.

In line with the objectives of the study, inclusion and exclusion criteria for the study group were established.

Inclusion criteria:

- an informed written consent to participate in the study protocol
- complete study documentation
- absence of any musculoskeletal pathologies

Exclusion criteria:

- lack of consent to the study
- incomplete documentation of physical exams
- a history of musculoskeletal pathologies (e.g., juvenile idiopathic arthritis, spondyloarthropathies, arthritis associated with infections, generalized lupus erythematosus, dermatomyositis, scleroderma, vasculitis)

### 2.2. Study Design

The study protocol was pursued in the posturology laboratory. The assessment of the plantar part of the foot in static conditions was pursued using an Italian-made PodoScan 2D FootCAD, fitted with a CCD converter with a cold cathode (1600 DPi). This technologically advanced device facilitates digital analysis of the plantar footprints and loads. The actual image of the plantar part of the foot is analyzed, so that its length, width, angles, and axes may be determined. During the test, the subjects stood on the device barefoot, the lower limbs upright, the upper limbs hanging along the body, the feet parallel. Examination of the feet was carried out under the load of their own weight.

Body height was measured using the SECA growth meter of German production 93/42/EEC, 2007/47/EC (measurement accuracy 0.01m). Body weight was assessed using a Tanita BC-418MA scale of Japanese manufacture 93/42/EEC Annex II (measurement accuracy ±0.1 kg). The morphological structure of the plantar part of the foot in static conditions was verified with the aid of a 2D podoscan (Sensor Medica, Rome, Italy). Foot loading in static conditions was assessed by FreeMed platform, operated by FreeStep Pro software (FreeMed, Sensor Medica, Rome, Italy, no.10806) [7,8].

The following indicators were assessed:

Sztriter–Godunow index KY—is the ratio of the length of the segment running through the shaded part of the metatarsal reflection (BC) to the length of the unshaded and shaded part (AC) KY = BC/AC [9,10,11].

Chippaux–Smirak index CSI—is the ratio of the minimum metatarsal distance to the maximum forefoot distance

CSI = i/S [5,12,13]

Staheli index SI—is the ratio of the minimum metatarsal distance to the maximum hindfoot distance

SI = AB/CD [13,14]

The classification of results was based on the following standards:

Sztriter–Godunow index: hollow foot 0.00–0.25; normal 0.26–0.45; reduced I° 0.46–0.49; reduced II° 0.50–0.75; flat 0.75–1.00 [9,10]

Chippaux–Smirak index: CSI < 25%—hollow foot; 25% < CSI < 45%—normal;

CSI > 45%—flat foot

Staheli index: 044–0.89—normal foot; SI < 0.44—hollow; SI > 0.89—flat [14]

**Figure 2:**
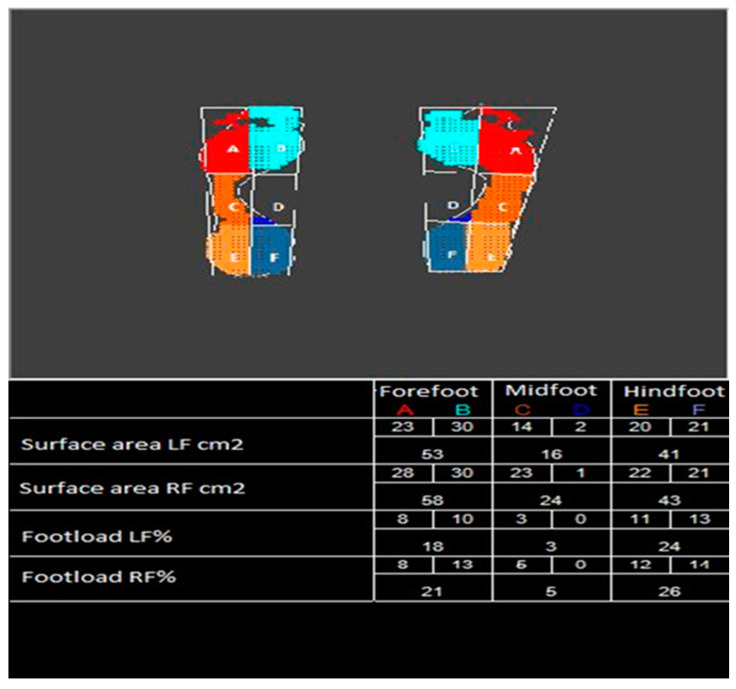
An example of the foot loading test outcome, under static conditions; demarcation of respective zones within the plantar part of the foot. Source: Own research material.

Static examination with the FreeMed platform presented the division of the foot into six zones: the forefoot is zone A and B, the metatarsal—zone C and D, the hindfoot zone E and F (Figure 2).

Division of the foot into respective zones:

A—lateral part of the forefoot

B—medial part of the forefoot

C—lateral part of the metatarsal

D—medial part of the metatarsal

E—lateral part of the hindfoot

F—medial part of the hindfoot (Figure 2).

When testing under the static conditions, the foot was divided into six zones, i.e., forefoot zones—A and B, metatarsal zones—C and D, and hindfoot zones—E and F (Figure 2).

### 2.3. Statistical Methods

Statistical analyses were carried out making use of the R programme v. 4.0.1.

The non-parametric Mann–Whitney–Wilcoxon test was applied to compare basic descriptive variables in the girls’ and boys’ groups, respectively.

All dependent variables relating to the load-bearing level in the respective foot zones were analyzed for normality of distribution. A Box–Cox transformation was developed with a view to modeling where the dependent variables boasted just these properties.

The loading of both feet, left and right, was included in the models. Considering, however, that the observations are not independent in this approach, the study participant was added on as a random effect. As several foot zones were assessed, the significance level was adjusted by making use of the Sidak correction for multiple comparisons. The results for the Box–Cox transformed variables were again transformed using an inverse transformation to acquire the final results. For the Staheli index and the Sztriter–Godunow index, flat feet were not considered in the loading analysis, in view of a small size of this particular group. The above-referenced dependences were considered statistically significant when the level of significance was *p* < 0.05.

## 3. Results

Table 2 presents the mean and SD from the indices under study.

Table 3 indicates the loading of respective parts of the foot.

Table 4 provides a breakdown of the feet, whilst taking into account the KY, SI, CSI indices. These indices are very strongly correlated with each other ρ > 0.84, *p* < 0.001. (ρ—Pearson correlation, *p*—level of significance).

**Figure 3:**
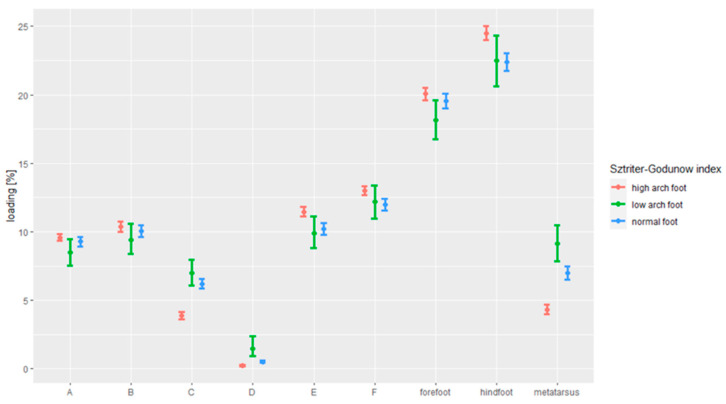
The dependence between foot loading under the static conditions and the Sztriter–Godunow index.

Figure 3 demonstrates the results of assessing the association between the foot loading paradigm and the foot arches, in line with the Sztriter–Godunow index.

Persons with elevated arches put more pressure on the forefoot (20.06%), as compared to the ones with the lowered arches (18.16%; *p* = 0.042).

Persons with normal feet (6.99%) put more pressure on the metatarsal than the ones with the elevated arches (4.34%; *p* < 0.001) and the ones with the lowered arches (9.13%; *p* = 0.003). Persons with the lowered arches (9.13%) put more pressure on the metatarsal than the ones with the elevated arches (4.34%; *p* < 0.001).

Persons with normal feet (22.40%) put less pressure on the hindfoot than ones with elevated arches (24.50%; *p* < 0.001).

Persons with normal feet (6.20%) put more strain on Zone C than the ones with the elevated arches (3.89%; *p* < 0.001). Also, the persons with the elevated arches (3.89%) put less pressure on Zone C than the ones with the lowered arches (6.99%; *p* < 0.001).

Persons with normal feet (0.53%) put more pressure on Zone D than the ones with the elevated arches (0.26%; *p* < 0.001), and also put less pressure on Zone D than the ones with the lowered arches (1.45%; *p* < 0.001). Persons with lowered arches (1.45%) put more pressure on Zone D than the ones with the elevated arches (0.26%; *p* < 0.001).

Persons with normal feet (10.21%) put less pressure on Zone E than the ones with the elevated arches (11.47%; *p* < 0.001).

Persons with normal feet (12.01%) put less pressure on Zone F than the ones with the elevated arches (13.00%; *p* = 0.001).

**Figure 4:**
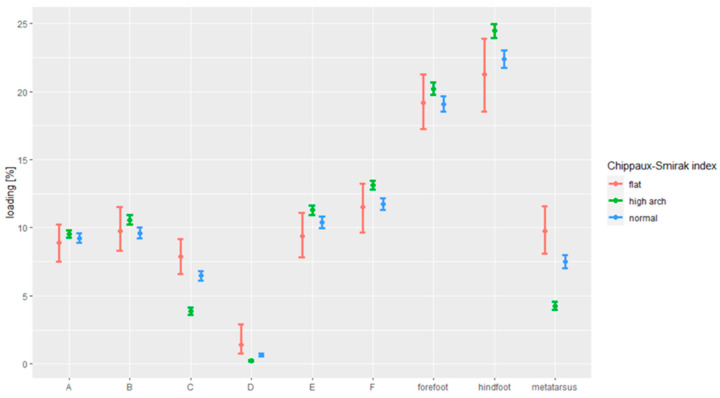
The dependence between foot loading under the static conditions and Chippaux–Smirak index.

Figure 4 demonstrates the results of the analysis of the correlation between the foot loading paradigm and the foot arching, in line with the Chippaux–Smirak index.

Persons with elevated arches (20.21%) put more pressure on the forefoot than the ones with normal feet (19.09%; *p* = 0.003).

Persons with elevated arches (4.26%) put less pressure on the metatarsal than the ones with normal feet (7.50%; *p* < 0.001) and flat feet (9.75%; *p* < 0.001). Persons with normal feet (7.50%) put less pressure on the metatarsal than the ones with flat feet (9.75%; *p* = 0.025).

Persons with high-arched feet (24.45%) put more pressure on the hindfoot than the ones with normal feet (22.38%; *p* < 0.001).

Persons with elevated arches (10.58%) put more pressure on Zone B than the ones with normal feet (9.60%; *p* = 0.001).

Persons with the elevated arches (3.86%) put less pressure on Zone C than the ones with normal feet (6.47%; *p* < 0.001). Persons with the high-arched feet (3.86%) put less pressure on Zone C than the ones with flat feet (7.86%; *p* < 0.001).

Persons with the elevated arches (0.24%) put less pressure on Zone D than the ones with normal feet (0.65%; *p* < 0.001). Persons with the high-arched feet (0.24%) put less pressure on Zone D than the ones with flat feet (1.41%; *p* < 0.001).

Persons with the high-arched feet (11.29%) put more pressure on Zone E than the ones with normal feet (10.41%; *p* = 0.009).

Persons with the elevated arches (13.13%) put more pressure on Zone F than the ones with normal feet (11.73%; *p* < 0.001).

**Figure 5:**
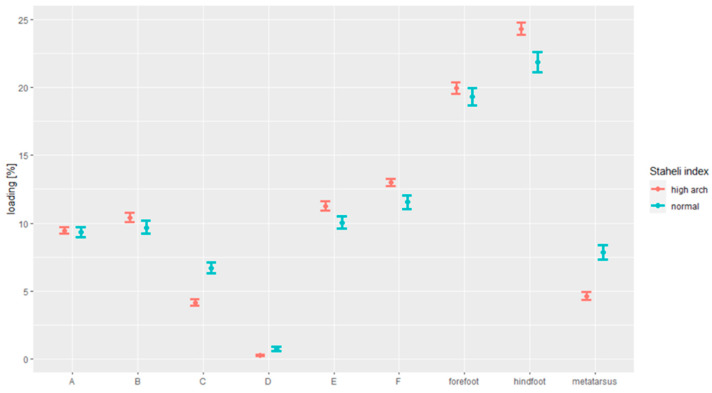
The dependence between the foot loading paradigm under the static conditions and Staheli index.

Figure 5 demonstrates the results of the analysis of the correlation between foot loading and foot arching, in line with the Staheli index.

Persons with the elevated arches (4.62%) put less pressure on the metatarsal than the ones with normal feet (7.83%; *p* < 0.001).

Persons with the elevated arches (24.33%) put more pressure on the hindfoot than the ones with normal feet (21.86%; *p* < 0.001).

Persons with the elevated arches (4.16%) put less pressure on Zone C than the ones with normal feet (6.70%; *p* < 0.001).

Persons with the elevated arches (0.28%) put less pressure on Zone D than the ones with normal feet (0.72%; *p* < 0.001).

Persons with the elevated arches (11.27%) put more pressure on Zone E than the ones with normal feet (10.05%; *p* < 0.001).

Persons with the elevated arches (13.00%) put more pressure on Zone F than the ones with normal feet (11.55%; *p* < 0.001).

No statistically significant associations were observed between the indices under study and gender and age.

## 4. Discussion

The principal purpose of this study consisted in establishing conclusively overall reliability and diagnostic accuracy of respective, select morphological variables of the foot, and their correlation with an individual foot loading paradigm, whilst making use of the KY, SI, and CSI indices as the key indicators in assessing the foot’s longitudinal arches. Evidence yielded throughout the study protocol gave the investigators sufficient grounds to believe that not only were all three indices strongly correlated, but also proved their reliability as accurate diagnostic tools.

One of the key links in the human biokinematic chain is made up by the foot. The correct morphological structure is reflected in its efficiency and performance. Any disorder in the actual structure and functionality of the foot may promote various dysfunctions in the other components of the locomotor system. Researchers highlight that foot arching abnormalities may account for pain in the foot, but also in the calf, knee, hip, and trunk joints. They also contribute to disorders in the gait pattern and impair balance [15,16,17]. Elevation or lowering of the foot’s longitudinal arch may ostensibly be a trivial, non-painful, almost invisible problem, even though its long-term consequences may well become quite dramatic. Effective assessment of overall functional capacity and morphological structure of the foot is therefore absolutely essential.

Diagnosing and subsequent categorization of the feet appears a truly daunting task. Apart from the investigator’s own experience, it also requires the application of methods that are repeatable, characterized by a low error rating, and may well be applied in population studies. In the present study, an attempt was made to establish whether the above-referenced requirements could be complied with by making use of the KY, SI, and CSI indices, in assessing the foot’s longitudinal arch, and whether this approach might subsequently be corroborated by the data supplied through the assessment of the foot loading paradigm. Own research indicates that making use of a podoscope coupled with a computer may actually facilitate an objective assessment. This approach may still be supplemented by the use of a ground reaction force platform which makes it possible to assess the pressure on the support plane within the respective zones of the foot.

There are reports corroborating the correlation between the foot loading paradigm and various foot defects [18,19]. On the other hand, there is a manifest scarcity of published studies attesting to the overall reliability and accuracy of making use of the indices assessing the foot’s longitudinal arch, and confirming its validity in assessing the foot loading paradigm in children.

Buldt et al. [18] highlight in their study that individuals affected by hollow feet tend to exert appreciably greater pressure on the forefoot, whereas those characterized by flatfootedness tend to do so on the foot’s metatarsal area. The investigators concluded that each foot defect accounts for exerting a different rate of pressure on the plantar part of the foot, characteristic of that particular defect only. Jung Su Lee et al. [20] also noted an increased metatarsal pressure when studying children affected by flatfootedness. Similar associations were established in the study by Kirmizi et al. [21]. Woźniacka et al. [15], while studying a group of 81 women, reported an increased forefoot pressure among those boasting elevated longitudinal arches in their feet. A structural change in the arch accounted for an asymmetrical lower limb loading and a resultant change in the shoulder girdle height alignment.

There are numerous studies [3,9,11,15] corroborating the overall pertinence of assessing the longitudinal foot arches. Diversity of testing methods applied for the task, in conjunction with there being no age-specific reference values, nor indeed any set diagnostic standards, make it rather hard to make an assessment of the foot’s longitudinal arches a fully credible process. The incidence of flatfootedness appears to pose an extra challenge. Discrepancies in the studies focused on assessing flatfootedness range from 4.7% to 75% [11,15,22,23,24]. Similar differences are notable with regard to assessing a hollow foot—ranging 9–66.55% [15,25]. The present study made use of four approaches in this assessment, with a view to establishing the overall reliability of the select indices under study. The results actually yielded indicated there was, in fact, very little difference in assessing the correlation between the longitudinal arch and the foot loading paradigm, when making use of the KY, SI, and CSI indices.

This comparison was as follows: normal arches in line with the KY index were encountered in 34.5% of the left feet, and in 39.9% of the right feet, in line with the SI index—in 25.6% of the left feet and 30.7% of the right feet, whereas in line with the CSI index—in 34.2% of the left feet and 38.7% of the right feet. With regard to the incidence of flatfootedness, in line with the KY index, it was established in a single left foot, when aided by the SI index—in a single left and a single right foot, when applying the CSI index—it was reported in 1.2% of the left feet and 2.7% of the right feet. Hollow feet—when aided by the KY index—in 61.6% of the left feet and 55.1% of the right feet, whereas the SI index attested to 74.1% of the left feet and 69% of the right feet, the CSI index indicated 64.6% of the left feet and 58.6% of the right feet.

All indices indicated an increased incidence of the hollow feet, both right and left ones. Numerous studies highlight that a change in foot loading paradigm is associated with an alteration in the structure of the longitudinal foot arch [19,26,27]. As evidenced by our own research, an increased loading in the hollow feet, as assessed by KY and SI CSI indices on the forefoot and hindfoot area, in zones B, E, F, is statistically significant. All three indices attested to an increased loading on the metatarsal, and zones C, D in the flat feet.

In the study of Žukauskas et al. [5], strong correlations of flatfootedness in children were reported between the SI and CSI indices and FPI-6. Similar correlations were established in the study by Zuil-Escobar et al. [3], whereby similarity between the foot assessment aided by the SI and CSI indices was established. Puszczałowska–Lizis [28], on the other hand, assessed a group of 130 students and found the Chippaux–Smirak index to be unreliable in the assessment of longitudinal arches.

In a study of students aged 20–28 years, the author proposes her own, self-devised index for assessing the longitudinal arch of the foot. By making use of the Clarke’s, Sztriter–Godunov’s, Chappaux–Smirak’s indices, along with her own, in assessing the actual height of the foot’s longitudinal arching of the foot, and subsequently presenting the study outcomes with the aid of factorial analysis, she concluded that by far the best index for that task was the one proposed by herself. In view of the low input values of the factor loadings for the Chappaux–Smirak index, the author considered this index to be of little use in assessing the longitudinal foot arching. This, however, remains in stark contrast to the evidence yielded by our own findings, as we regard the SI, CSI, and KY indices, in conjunction with the foot’s plantar pressure analysis, as by far the best suited ones for this particular assessment task.

The effect of age and gender on the loading of the plantar part of the foot was noted in the study by Demirbüken et al. [29]. The investigators studied 524 individuals, aged 11–14 years, and concluded that gender- and age-dependent foot loading changes attested to some potential risk factors for foot abnormalities. The study did not establish any correlations between gender and the foot loading paradigm. Similar results were yielded by our own study.

The results yielded by the present study give sufficient grounds to believe that the KY, SI, and CSI indices, and their correlation with an individual foot loading paradigm, offer a satisfactory reliability, whereas any foot defects diagnosed through their application are well reflected by the key foot loading variables.

The authors believe that comprehensive appreciation of the actual linkage between an individual foot loading paradigm and different methods of assessing the foot’s longitudinal arches, is essential in terms of effective prevention policies, whereas their own findings may well offer specific pointers in mapping out such policies, with a view to securing them a nationwide appeal.

## 5. Conclusions

1. Flatfootedness was not found a common deformity among children and adolescents.
2. Statistically significant linkage, as well as a strong correlation, was established for the Staheli, Chippau–-Smirak, and Sztriter–Godunow indices.
3. Consequently, the Staheli, Chippaux–Smirak, and Sztriter–Godunow indices were deemed the most reliable indicators in assessing the foot’s longitudinal arches.

## Figures and Tables

**Table 1:** Basic characteristics of the study group.

Variable	Girls (n = 165) Mean ± SD	Min–Max	Boys (n = 171) Mean ± SD	Min–Max	Z	*p*
Body mass [kg]	42.91 ± 11.17	25–77	45.21 ± 12.08	25–85	−1.568	0.117
Height [cm]	150 ± 10	128–172	151 ± 12	130–183	−0.251	0.802
BMI	18.68 ± 3.25	13.3–28.6	19.46 ± 3.33	13.2–29.1	−2.122	0.034
Age	11.47 ± 1.53	10–15	11.42 ± 1.45	10–15	0.150	0.881

Mean—arithmetic mean, SD—standard deviation, Z—statistical values of the Mann–Whitney–Wilcoxon test for two independent samples, BMI—body mass index, *p*—significance level.

**Table 2:** Mean and SD from the respective indices.

Foot	Sztriter–Godunow Index		Chippaux–Smirak Index		Staheli Index	
	Mean	SD	Mean	SD	Mean	SD
Right	0.290	0.155	21.693	12.034	0.349	0.192
Left	0.272	0.157	20.547	11.356	0.332	0.186

**Table 3:** Mean and SD of the foot loading.

Loading of Respective Foot Zones	Foot			
	Right		Left	
	Mean	SD	Mean	SD
Loading of the forefoot %	20.377	3.922	19.695	4.244
Loading of the metatarsal %	5.868	3.517	5.919	3.549
Loading of the hindfoot %	22.201	4.898	24.820	4.812
Loading of foot zone A %	9.901	2.003	8.605	3.006
Loading of foot zone B %	10.476	2.681	11.090	4.571
Loading of foot zone C %	5.296	2.893	4.805	2.605
Loading of foot zone D %	0.572	1.079	1.114	1.355
Loading of foot zone E %	10.114	2.846	12.404	3.676
Loading of foot zone F %	12.087	2.803	12.416	4.320

**Table 4:** Classification of the feet in terms of KY, SI, and CSI indices.

Foot	Index KY				Index SI				Index CSI			
	Left Foot		Right Foot		Left Foot		Right Foot		Left Foot		Right Foot	
	N	%	N	%	N	%	N	%	N	%	N	%
Normal	116	34.5	134	39.9	86	25.6	103	30.7	115	34.2	130	38.7
Flatfoot	1	0.3	0	0	1	0.3	1	0.3	4	1.2	9	2.7
Low arch Foot	12	3.6	17	5.1	-	-	-	-	-	-	-	-
High arch foot	207	61.6	185	55.1	249	74.1	232	69	217	64.6	197	58.6

KY-Sztriter-Godunow index; SI-Staheli index (SI); CSI-Chippaux-Smiraka index.

## Data Availability

The datasets generated and/or analyzed during the current study are available from the Corresponding Author upon reasonable request.
